# Alcohol-soluble Star-shaped Oligofluorenes as Interlayer for High Performance Polymer Solar Cells

**DOI:** 10.1038/srep17329

**Published:** 2015-11-27

**Authors:** Yang Zou, Zhicai He, Baofeng Zhao, Yuan Liu, Chuluo Yang, Hongbin Wu, Yong Cao

**Affiliations:** 1 Hubei Collaborative Innovation Center for Advanced Organic Chemical Materials, Hubei Key Lab on Organic and Polymeric Optoelectronic Materials, Department of Chemistry, Wuhan University, Wuhan 430072, China; 2 Institute of Polymer Optoelectronic Materials and Devices, State Key Laboratory of Luminescent Materials and Device, South China University of Technology, Guangzhou 510640, China

## Abstract

Two star-shaped oligofluorenes with hexakis(fluoren-2-yl)benzene as core are designed and sythesized for interfacial materials in polymer solar cell. Diethanolamino groups are attached to the side chain of fluorene units for T0-OH and T1-OH to enable the alcohol solubility, and additional hydrophobic *n*-hexyl chains are also grafted on the increased fluorene arms for T1-OH. In conventional device with PCDTBT/PC_71_BM as active layer, a 50% enhanced PCE is obtained by incorporating T0-OH and T1-OH as the interlayer compared with device without interlayer. By optimizing the active material with PTB7 and with the inverted device structure, a maximum PCE of 9.30% is achieved, which is among the highest efficiencies for PTB7 based polymer solar cells. The work function of modified electrode, the surface morphology and the suraface properties are systematically studied. By modifying the structures of the star-shaped molecules, a balance between the hydrophobic and hydrophilic property is finely tuned, and thus facilitate the interlayer for high performance of PSCs.

Polymer-fullerene bulk heterojunction solar cells (PSCs) have attracted extensive attention due to their advantages, such as low cost of materials, ease of fabrication, light weight physical feature and easy for integration into flexible devices with large area^1^,^2^,^3^,^4^. The power conversion efficiency (PCE) has grown rapidly in recent years and exceeding 10% of PCE has been reported^5^,^6^,^7^,^8^. Generally, there are several methods to enhance the PCE of PSCs, such as optimizing the chemical structure of materials for the active layer with better absorption and energy level characteristics^2^,^9^, inserting an interlayer between electrode and active layer^10^,^11^,^12^, adding additives or modifying device with inverted or tandem structure^13^,^14^. Among these methods, the interlayers are essential for achieving highly efficient PSCs as they allow for more efficient collection and extraction of charges even in a very low dosage^15^,^16^.

A large number of suitable interfacial materials for PSCs have been reported, such as metal complexes^17^, organic-inorganic composite^18^,^19^,^20^, graphene derivatives^21^,^22^, nanoparticle clusters^23^, and most importantly, alcohol-soluble conjugated polymers^10^. Alcohol-soluble conjugated polymers had already been used as the interlayers for organic light emitting diodes (OLEDs) in recently years^24^,^25^,^26^. They have also been proved to be excellent interfacial materials for PSCs as they possess good film-forming ability, good solution-processability, and can simultaneously enhance the open-circuit voltage (*V*_oc_), short-circuit current density (*J*_sc_) and fill factor (*FF*), resulting in a significantly enhanced PCE. Up to date, a large number of alcohol-soluble conjugated polymers with linear or three dimensional structures have been used as the interlayers in PSCs, and satisfied device performances have been achieved^27^,^28^. Although these alcohol-soluble polymers were reported as the interlayers for PSCs, the structure-performance relationship has not been deeply discussed yet. Since these interfacial materials are polymers, the interlayer performances are probably dependent on the molecule weight, polydispersity index (PDI) and defects of the polymer materials, which may make us difficult to get deep understanding of the role of the interlayers.

Star-shaped molecules have been used in various of optoelectronic devices^29^,^30^,^31^,^32^. They can be regarded to be intermediates of small molecules and polymers, as a consequence of which, they possess advantages of both, such as good solubility, excellent film-forming ability, high stability and precise energy levels. What’s more, their properties are completely reproducible due to their well-defined structures. Recently, we reported a series of star-shaped molecules with hexakis(fluoren-2-yl)benzene (HFB) core^33^,^34^,^35^. They processed good film-forming ability, high thermal stability and have been proved to be good optoelectronic materials for OLEDs.

Herein, aiming to develop new interfacial materials for PSCs, we designed and synthesized two alcohol-soluble star-shaped molecules with HFB as a core and fluorene units as the arms, namely T0-OH and T1-OH (see Fig. 1). For both molecules, diethanolamino groups are attached to the side chain of fluorene units to enable the alcohol solubility, and for T1-OH, additional hydrophobic *n*-hexyl chains are also grafted on the side chain of the fluorene arms. It is supposed that these alcohol soluble HFB-cored star-shaped oligofluorenes have several advantages as the interlayers in PSCs. First of all, the diethanolamino groups on the 9-position of the fluorene arms, on one hand, can effectively reduce the work function (*W*_*F*_) of the electrode and benefit charge extraction^36^,^37^. On the other hand^34^, they can provide material with alcohol-solubility, which is necessary for fabricating the solution-processable device with low cost. Secondly, as star-shaped molecules, they possess good film-forming ability, which is essential for fabricating device with good homogeneity and high efficiency^38^. Thirdly, they possess high optical transparency in the visible and near-infrared region due to high energy gap resulted from the highly twisted backbone of HFB core, which allows photons to reach the active layer efficiently^34^. Finally, comparing to other polymers-based interlayer which consists of a mixture with different molecule weight, these compounds possess precise structure, and their properties can be precisely fine-tuned by chemical structure modification, which allows us to make deep understanding of the relationship between the structure and the performance of the interfacial material for PSCs. With these desired properties, we fabricated PSCs with T0-OH and T1-OH as the interlayer, and a blend of [6,6]-phenyl C_71_ butyric acid methyl-ester (PC_71_BM) and poly[N-9″-hepta-decanyl-2,7-carbazole-alt-5,5-(4′,7′-di-2-thienyl-2′,1′,3′-benzothiadiazole)] (PCDTBT) as the active layer to evaluate the interfacial modification performance. The results proved these alcohol-soluble star-shaped compounds to be good interfacial materials with a 50% enhanced PCE. By optimizing the active material with poly[[4,8-bis[(2-ethylhexyl)oxy]benzo[1,2-b:4,5-b’]dithiophene-2,6-diyl][3-fluoro-2-[(2-ethylhexyl)carbonyl]thieno[3,4-b]thiophenediyl]] (PTB7) and with the inverted device structure, a maximum PCE of 9.30% was achieved. Our research provide new ways to further precise chemical structural optimization of the interfacial materials.

## Results and Discussion

The star shaped oligofluroenes were synthesized by modified convergent core-creating approach (Supplementary Figure S1). The two key intermediates of 1,2-bis(oligofluoren-2-yl)ethyne were synthesized via palladium catalyzed one-pot Sonagashira coupling and Suzuki coupling, with bromohexyl chains on the fluorene units. The HFB core was constructed through cobalt catalyzed [2 + 2 + 2] cyclotrimerization reaction, and then the bromohexyl groups were transformed to diethanolamino groups by a simple nucleophilic substitution reaction to afford the final products of T0-OH and T1-OH in high yield. The intermediates and the desired products were fully verified by ^1^H and ^13^C NMR spectroscopy, elemental analysis, MALDI-TOF mass spectrometry (see Supplementary Information). Both T0-OH and T1-OH show good solubility in oxygen-containing solvents, such as methanol, ethyl acetate and tetrahydrofuran, but they are almost insoluble in hexane, dichloromethene, dichlorobenzene at ambient temperature. The solubility feature makes them easy to purify and suitable for fabricating PSCs via solution-processable method.

The photophysical and electrochemical properties of T0-OH and T1-OH were characterized by UV-vis and cyclic voltammetry (CV) experiments (Supplementary Figure S2). The absorption spectra of T0-OH and T1-OH in film are identical to those in methanol solution. The maximum absorption wavelengths are 315 and 342 nm, and the optical bandgaps are calculated to be 3.65 and 3.30 eV for T0-OH and T1-OH, respectively, which are very similar to their diethanolamino-absent analogues^34^. No absorption in visible or infrared region was observed for either compound, providing optical transparency for efficient light harvesting when they are incorporated in PSCs as the interlayers. As the electrochemical properties are mainly defined by their fluorene arms, each compound shows identical CV curve compared to its diethanolamino-absent analogue^34^. The absorption data, the highest/lowest occupied molecular orbital energies (HOMO/LUMO) are summarized in Supplementary Table S1.

**Figure 2:**
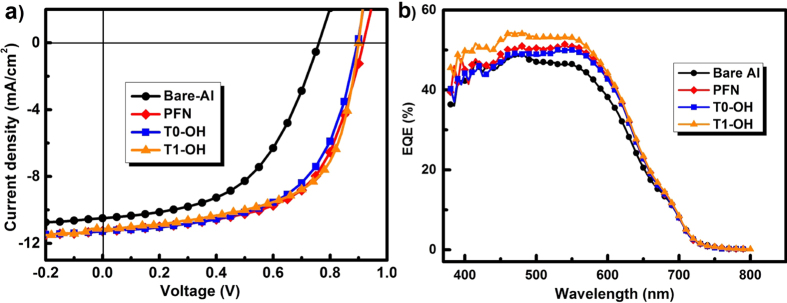
(**a**) *J-V* characteristics and (**b**) external quantum efficiency (EQE) spectra of the PSCs based on PCDTBT:PC_71_BM as the active layer, with and without the interlayers.

To evaluate the performance of these alcohol-soluble star-shaped compounds as the interlayer in PSCs, we initially fabricated the devices with the configurations of ITO/PEDOT:PSS/PCDTBT:PC_71_BM/T0-OH or T1-OH/Al. Besides, control devices without the T0-OH/T1-OH interlayer, or with poly[(9,9-bis(3’-(N,N-dimethylamino)propyl)-2,7-fluorene)-*alt*-2,7-(9,9-dioctylfluorene)] (PFN)^10^ as interlayer were also fabricated. The current density-voltage (*J-V*) characteristics are shown in Fig. 2, and the *J*_sc_, *V*_oc_, *FF* and PCE data are summarized in Table 1. The devices with the interlayer of T0-OH or T1-OH display ca. 50% enhancement of PCE (5.95%~6.20%) compared with the control device without the interlayer (bare-Al cathode), owing to the simultaneously enhanced *J*_sc_, *V*_oc_ and *FF*, while the PFN based devices also shows a high average PCE of 6.00%.

The T0-OH and T1-OH interlayer also work very well for other promising donor polymer: PC_71_BM system. For instance, when the PCDTBT:PC_71_BM blend was replaced by PTB7:PC_71_BM, in which PTB7 possesses a low bandgap (~1.6 eV) and intense absorption throughout the region of the greatest photon flux in the solar spectrum^2^,^39^.

**Figure 3:**
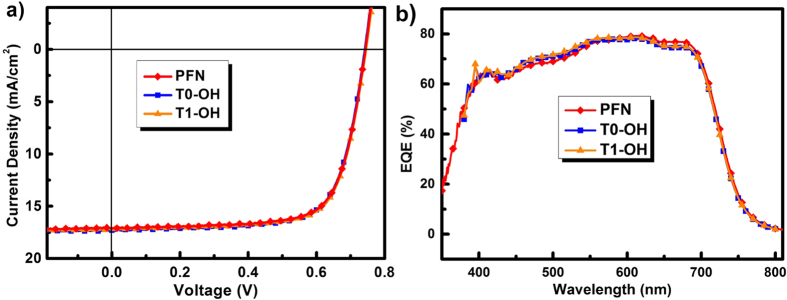
(**a**) *J-V* characteristics and (**b**) EQE spectra of the inverted PSCs with PTB7:PC_71_BM as the active layer and **T0-OH**, **T1-OH** or PFN as the cathode interlayer, with a device structure of ITO/interlayer/PTB7:PC_71_BM/MoO_3_/Al.

As showed in Fig. 3 and Table 2, all the devices with either T0-OH and T1-OH interlayer exhibited excellent performance, with an average PCE around 9.1% and a maximal value of 9.30%, which is comparable or even slightly higher than those of devices with PFN interlayer (with an average PCE of 8.99% and a maximal value of 9.19%, respectively, see Table 2).

**Figure 4:**
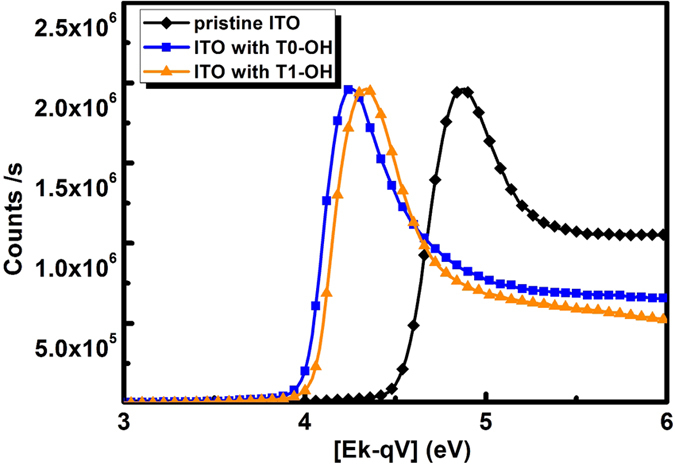


One of the reasons for the enhanced performance of PSCs can be attributed to the formation of interfacial dipoles between ITO and interlayer and the consequent decrease in the work function of the ITO substrate, as a result of the interaction between the amino groups on the fluorene side chains and ITO^39^. Indeed, as demonstrated by X-ray photoelectron spectroscopy (XPS) in Fig. 4, the work function decreased from 4.7 eV (for bare ITO) to 4.0 and 4.1 eV for T0-OH and T1-OH, respectively. The modified ITO substrates with lower work function well match the LUMO level of PC_71_BM, which allows for better electron extraction^36^,^40^,^41^. Previously, various possible origins for the interfacial dipole between organic/metal interface have been proposed^42^, including surface dipole due to the redistribution of surface electron density tail upon molecular adsorption, charge transfer between the adsorbed molecules and the substrate, or the alignment of polar diethanolamino groups at the side chain. Given the intense polar diethanolamino groups in T0-OH and T1-OH, we conclude interfacial dipole between the interlayer and electrode could be the origin of enhanced device performance in the devices.

For the inverted devices with PTB7:PC_71_BM as the active layer, it is noteworthy that T1-OH shows a slightly higher PCE (9.30%) than T0-OH (9.22%). It is recalled that T1-OH also exhibits the higher PCE (6.20%) than T0-OH (5.95%) for the conventional devices with PCDTBT:PC_71_BM as the active layer. To understand the relationship between the structure diversity and different device performance, we further investigated the surface morphologies and wetting properties by using the interfacial materials.

**Figure 5:**
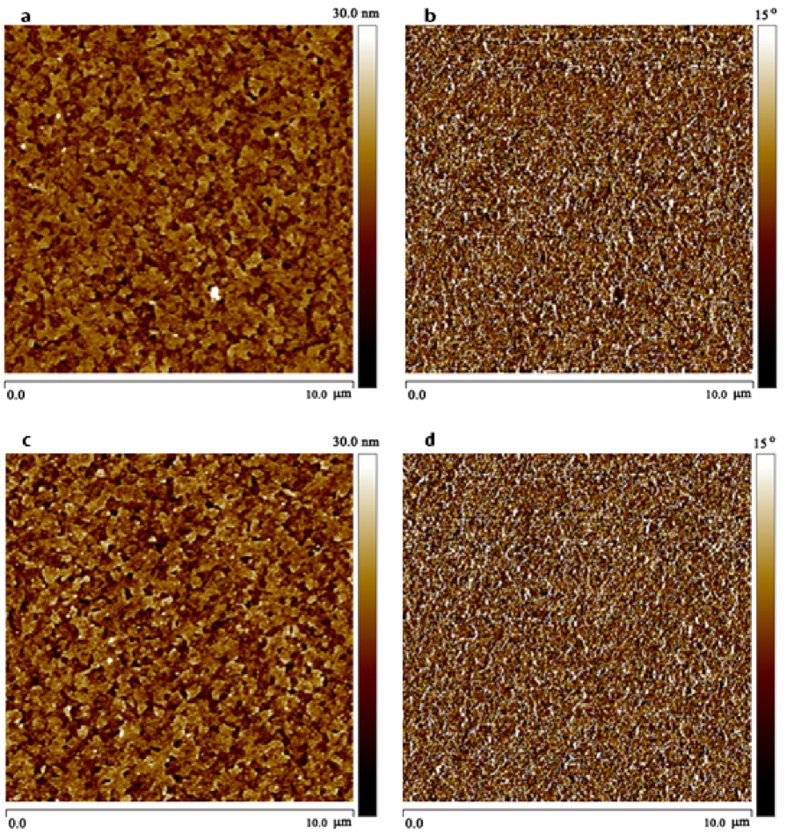


Figure 5 shows the surface morphologies obtained by atomic force microscopy (AFM). After deposition of T0-OH or T1-OH on ITO, both surfaces are homogeneous and with a root mean square (rms) roughness of 3.70 nm (Fig. 3a) or 4.50 nm (Fig. 3c), respectively. The rough surface was believed to offer better interaction between bulk heterojunction (BHJ) composite layer and ITO cathode, and possibly more effective electron injection was achived by a larger contact area between BHJ and cathode^43^.

**Figure 6:**
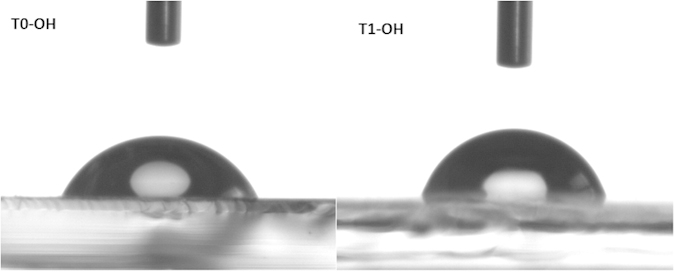


The wetting properties of the cathodic interlayers were also tested. Measurements of the water contact angle were performed on the surface of T0-OH and T1-OH atop of ITO substrate. As shown in Fig. 6, the water contact angle of T1-OH (75°) is larger than that of T0-OH (64°), indicating that the surface of T1-OH is more hydrophobic than that of T0-OH. The hydrophobic surface of T1-OH layer will benefit the intimate contact with the active layer which could better spread out from an organic solution, and consequently get a better morphology and interpenetrating networks for BHJ composite^44^. The structure of T1-OH combines the long alkyl chains in the inner fluorene arms and the diethanolamino groups in the outer fluorene arms, and therefore it may get a balance between the hydrophobic and hydrophilic properties. The hydrophilic feature would benifit to its interaction with ITO in the inverted devices and metal cathode in the conventional devices, and the hydrophobic chacracteristic would facilitate its contact with polymeric active layer in both inverted and conventional devices.

## Conclusion

In conclusion, we developed two alcohol soluble star-shaped oligofluorenes of T0-OH and T1-OH, and demonstrated that they are excellent interfacial materials for PSCs. The conventional devices with T0-OH/T1-OH as the interlayer and PCDTBT as the donor material achieved the highest PCE of 6.20%. The inverted devices with T0-OH/T1-OH as the interlayer and PTB7 as the donor material achieved the highest PCE of 9.30%, which is among the highest efficiencies for PTB7 based PSCs. We noted that the T1-OH based devices showed higher efficiencies than T0-OH based ones for both inverted and conventional devices. We can modify the structures of the star-shaped molecules to tune a balance between the hydrophobic and hydrophilic properties, and therefore optimize the interlayer for high performance of PSCs. This work reveals that the precise molecular structures, the reproducibility, the good solution processability and the designing flexibility of the star-shaped molecules could open a new room for the development of interfacial materials.

## Methods

Device fabrication and measurement: Electron donor material PCDTBT, PTB7 and electron acceptor PC_71_BM were purchased from 1-material Inc and Aldrich, respectively, and used as received. poly(3,4-ethylenedioxythiophene): poly(styrenesulfonate) (PEDOT:PSS) (Clevios P PVP AI 4083) was obtained from H.C. Starck Clevios. The inverted device structure was ITO/interlayer/PTB7:PC_71_BM(1:1.5 by weight)/MoO_3_/Al and the conventional device structure was ITO/PEDOT:PSS/PCDTBT:PC_71_BM(1:4 by weight)/interlayer/Al. For conventional devices, a 40-nm-thick PEDOT:PSS anodic buffer layer was spin-cast on the ITO substrate, then dried in a vacuum oven at 80 °C overnight. The PCDTBT:PC_71_BM blend active layer, with a nominal thickness of 80 nm (with a variation of 20 nm over the entire film), was prepared by spin-coating a mixed solvent of chlorobenzene/1,8-diiodoctane (97:3% by volume) solution (concentration, 25 mg ml^−1^ ) at 1,000 r.p.m. for 2 min. The interlayer material was dissolved in methanol in the presence of a small amount of acetic acid (2 μl ml^−1^) and its solution (concentration, 2 mg ml^−1^) was spin-coated on top of the obtained active layer to form a thin interlayer. A 100 nm Al layer were subsequently evaporated through a shadow mask and form a top cathode. For inverted devices, the interlayer material was spin-coated on top of the precleaned ITO substrate, following by the spin-coating of the PTB7:PC_71_BM active layer, and the thermal deposition of a 10 nm MoO_3_ layer and a 100 nm Al layer as top anode. PCE values were determined from *J–V* curve measurement (using a Keithley 2400 sourcemeter) under 1 sun, AM 1.5G (air mass 1.5 global) spectrum from a solar simulator (Oriel model 91192) (100 mW cm^−2^). Masks made from laser beam cutting technology with well-defined area size of 16.00 mm^2^ were attached to define the effective area for accurate measurement. All of the masked and unmasked tests gave consistent results with relative errors within 5%. Solar simulator illumination intensity was determined by a monocrystal silicon reference cell (Hamamatsu S1133, with KG-5 visible color filter) calibrated by the National Renewable Energy Laboratory (NREL). The theoretical Jsc obtained by integrating the product of the EQE with the AM 1.5G solar spectrum perfectly agreed with the measured value to within 3%. The spectral mismatch factors (M) were calculated according to standard procedure, and M value of 1.03 was used to obtain correct photocurrent and efficiency for PCDTBT and PTB7 based devices, respectively. The tapping-mode AFM images were obtained by using a scanning probe microscope (Dimension3100V). XPS studies were performed on a Thermo-VG Scientific ESCALAB 250 photoelectron spectrometer using a monochromated AlKa (1,486.6 eV) X-ray source. All recorded peaks were corrected for electrostatic effects by setting the C−C component of the C1s peak to 284.8 eV. The base pressure in the XPS analysis chamber was <2 × 10^−9^ mbar. Contact angle measurements were performed on a video-based optical contact angle measuring instrument (OCA20, Dataphysics Corp.).

## Additional Information

**How to cite this article**: Zou, Y. *et al.* Alcohol-soluble Star-shaped Oligofluorenes as Interlayer for High Performance Polymer Solar Cells. *Sci. Rep.* **5**, 17329; doi: 10.1038/srep17329 (2015).

## Supplementary Material

Supplementary Information

## Figures and Tables

**Figure 1:**
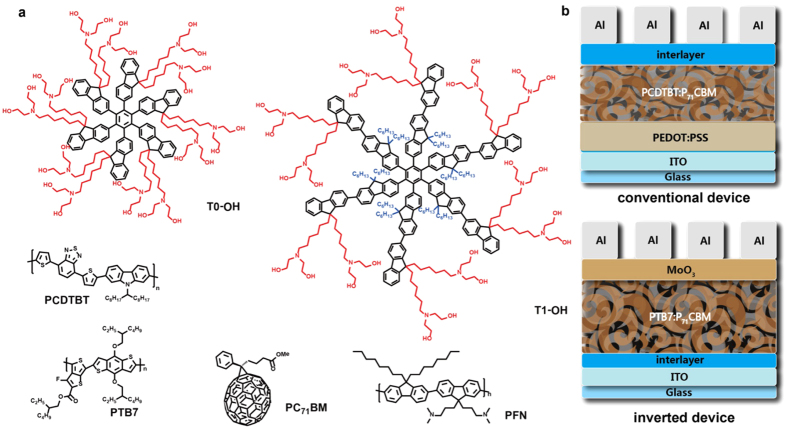
(**a**) Chemical structures of T0-OH, T1-OH and photoactive materials used in our study; (**b**) Devices configuration of conventional and inverted polymer solar cell.

**Table 1:** Photovoltaic properties of the PSCs with PCDTBT as active material and various interlayers under AM 1.5G illumination (100 mW cm^−2^), the average values were calculated from 10 individual devices fabricated under the same conditions with standard deviation for the measurements.

Interlayer	*V*_oc_ (V)	*J*_sc_ (mA cm^−2^)	*FF* (%)	PCE_av_ (PCE_MAX_) (%)
None	0.753 ± 0.004	10.3 ± 0.3	51.8 ± 1.8	4.01 ± 0.18 (4.22)
PFN	0.906 ± 0.008	10.96 ± 0.24	60.5 ± 0.6	6.00 ± 0.16 (6.17)
T0-OH	0.896 ± 0.005	10.98 ± 0.21	59.6 ± 1.4	5.89 ± 0.16 (6.07)
T1-OH	0.902 ± 0.012	11.00 ± 0.27	61.7 ± 1.1	6.13 ± 0.06 (6.20)

The device parameters of the best device are included in the parentheses for comparison.

**Table 2:** Photovoltaic properties of the inverted PSCs with PTB7 as active material and various interlayers under AM 1.5G illumination (100 mW cm^−2^), the average values were calculated from 10 individual devices fabricated under the same conditions with standard deviation for the measurements.

Interlayer	*V*_oc_ (V)	*J*_sc_ (mA cm^−2^)	*FF* (%)	PCE_av_ (PCE_MAX_) (%)
PFN	0.749 ± 0.002	17.08 ± 0.16	70.2 ± 1.2	8.99 ± 0.124 (9.19)
T0-OH	0.748 ± 0.005	17.18 ± 0.08	70.3 ± 1.2	9.05 ± 0.16 (9.22)
T1-OH	0.753 ± 0.005	17.20 ± 0.007	70.5 ± 1.0	9.14 ± 0.18 (9.30)

The device parameters of the best device are included in the parentheses for comparison.
