# Supplementary material for: Alcohol-soluble Star-shaped Oligofluorenes as Interlayer for High Performance Polymer Solar Cells
Source: Sci Rep. 2015 Nov 27;5:17329. doi: 10.1038/srep17329 (PMC4661482; doi:10.1038/srep17329)
Supplement: Supplementary Information [file srep17329-s1.doc]

Alcohol-soluble Star-shaped Oligofluorenes as Interlayer for High Performance Polymer Solar Cells

**Yang Zou^1+^, Zhicai He^2+^, Baofeng Zhao^2^, Yuan Liu^1^, Chuluo Yang^1*^, Hongbin Wu^2*^ and Yong Cao^2^**

^1^Hubei Key Lab on Organic and Polymeric Optoelectronic Materials, Department of Chemistry, Wuhan University, Wuhan 430072, China.

^2^Institute of Polymer Optoelectronic Materials and Devices, State Key Laboratory of Luminescent Materials and Device, South China University of Technology, Guangzhou 510640, China.

**General information**

^1^H NMR and ^13^C NMR spectra were measured on a MECUYR-VX300 spectrometer. Elemental analyses of carbon, hydrogen, and nitrogen were performed on a Vario EL III microanalyzer. EI mass spectra were measured on a ZAB 3F-HF mass spectrophotometer. MALDI-TOF mass spectra were performed on Bruker BIFLEX III TOF mass spectrometer. UV-vis absorption spectra were recorded on a Shimadzu UV-2500 recording spectrophotometer. Cyclic voltammetry (CV) was carried out in THF (oxidation scan) at room temperature with a CHI voltammetric analyzer. *n*-Bu_4_PF_6_ (0.1 M) was used as the supporting electrolyte. The conventional three-electrode configuration consists of a platinum working electrode, a platinum wire auxiliary electrode, and an Ag wire pseudoreference electrode with ferrocenium-ferrocene (Fc^+^/Fc) as the internal standard. Cyclic voltammograms were obtained at a scan rate of 100 mV s^-1^. The onset potential was determined from the intersection of two tangents drawn at the rising and background current of the cyclic voltammogram.

**Materials preparation**

Unless otherwise indicated, all starting materials were obtained from commercial suppliers and were used without further purification. Solvents for synthesis were purified by routine procedure and distilled under dry argon prior to use. 2-iodo-9*H*-fluorene[^1^](#_ENREF_1), 1,2-bis(7-bromo-9,9-dihexyl-9*H*-fluoren-2-yl)ethyne[^2^](#_ENREF_2), and 2-(9,9-bis(6-bromohexyl)-9*H*- fluoren-2-yl)-4,4,5,5-tetramethyl-1,3,2-dioxaborolane[^3^](#_ENREF_3) were prepared according to the literature.

**Figure S1.** synthetic routes of the compound.

***1,2-di(9H-fluoren-2-yl)ethyne (1).*** To a mixture of 2-iodo-9*H*-fluorene (4.38 g, 15 mmol), CuI (0.15 g, 0.75 mmol) and Pd(PPh_3_)_4_ (0.32 g, 0.45 mmol) were added degassed THF (75 mL) and 1,8-diazabicyclo[5.4.0]undec-7-ene (DBU) (13.5 mL). The mixture was stirred for 5 minutes under argon, then ethynyltrimethylsilane (1.05 mL, 7.4 mmol) and water (0.2 mL, 11 mmol) was quickly added. The mixture was sealed up and stirred for 24 h at room temperature. The resulting mixture was poured into water and the solid was collected by filtration. Then the crude product was boiled three times under chloroform to remove the by-product. Then the pale yellow product was collected by filtration. The crude product was used without further purification. MS (EI, m/z): [M]^+^ Calcd for C_28_H_18_: 354.1. Found: 354.2.

***1,2-bis(9,9-bis(6-bromohexyl)-9H-fluoren-2-yl)ethyne (2).*** 1,2-di(9*H*-fluoren-2-yl)ethyne (1.77 g, 5 mmol) was added batch-wise to a mixture of aqueous sodium hydroxide (100 mL, 50%), tetrabutylammonium bromide (0.33 g, 1 mmol), and 1, 6-dibromohexane (24.4 g, 100 mmol) at 75 °C. After 12 h, the mixture was cooled to room temperature. After extraction with CH_2_Cl_2_, the combined organic layers were washed successively with water, aqueous HCl (1 M), water, and brine and then dried over Na_2_SO_4_. After removal of the solvent and the excess 1,6-dibromohexane under reduced pressure, the residue was purified by silica gel column chromatography using hexane/chloroform (3:1, v/v) as the eluent to obtain the product as white powder (yield: 53%). ^1^H NMR (300 MHz, CDCl_3_, *δ*): 7.64-7.59 (m, 4H), 7.51-7.43 (m, 4H), 7.28-7.26 (m, 6H), 3.21 (t, *J* = 6.6 Hz, 8H), 1.92 (t, *J* = 8.1 Hz, 8H), 1.63-1.54 (m, 8H), 1.17-1.10 (m, 8H), 1.07-0.96 (m, 8H), 0.58-0.51 (m, 8H). ^13^C NMR (75 MHz, CDCl_3_, *δ*): 150.87, 150.69, 141.63, 140.69, 130.95, 127.91, 127.33, 126.11, 123.06, 121.86, 120.34, 120.03, 90.83, 55.28, 40.52, 34.26, 32.91, 29.32, 28.05, 23.77. MS (MALDI-TOF, m/z): [M]^+^ Calcd for C_52_H_62_Br_4_: 1006.2. Found: 1006.1. Anal. Calcd for C_52_H_62_Br_4_: C 62.04, H 6.21; Found: C 62.39, H 5.92.

***1,2,3,4,5,6-hexakis(9,9-bis(6-bromohexyl)-9H-fluoren-2-yl)benzene (3).*** To a mixture of 1,2-bis(9,9-bis(6-bromohexyl)-9H-fluoren-2-yl)ethyne (3.0 g, 3 mmol) and dicobaltoctacarbonyl (52 mg, 0.15 mmol) was added degassed 1,4-dioxane (60 mL). The solution was refluxed under argon for 24 h. The resulting mixture was poured into water, and the solid was collected by filtration. Then the crude product was purified by column chromatography using hexane/chloroform (3:1, v/v) as eluent to obtain the product as white powder (yield: 50%). ^1^H NMR (300 MHz, CDCl_3_, *δ*): 7.71-7.69 (m, 12H), 7.58-7.54 (m, 12H), 7.36-7.34 (m, 18H), 3.29 (t, *J* = 6.9 Hz, 24H), 2.00 (t, *J* = 8.4 Hz, 24H), 1.71-1.61 (m, 24H), 1.24-1.13 (m, 24H), 1.10-1.03 (m, 24H), 0.64-0.58 (m, 24H). ^13^C NMR (75 MHz, CDCl_3_, *δ*): 150.47, 149.24, 141.21, 140.09, 138.49, 129.68, 126.98, 126.78, 126.48, 122.79, 119.85, 118.01, 54.77, 40.57, 34.40, 33.12, 29.51, 28.09, 23.62. MS (MALDI-TOF, m/z): [M]^+^ calcd for C_156_H_186_Br_12_: 3019.5, found: 3018.0; Anal. calcd for C_52_H_62_Br_4_: C 62.04, H 6.21;Found: C 62.40, H 6.33.

***1,2-bis(9',9'-bis(6-bromohexyl)-9,9-dihexyl-9H,9'H-[2,2'-bifluoren]-7-yl)ethyne (4)***. To a mixture of 1,2-bis(7-bromo-9,9-dihexyl-9*H*-fluoren-2-yl)ethyne (1.70 g, 2 mmol) and 2-(9,9-bis(6-bromohexyl)-9H-fluoren-2-yl)-4,4,5,5-tetramethyl-1,3,2-dioxaborolane (3.10 g, 5 mmol), Pd(PPh_3_)_4_ (0.15 g, 0.2 mmol) and K_2_CO_3_ (1.38 g, 10 mmol) were added degassed THF (20 mL) and water (5 mL). The mixture was refluxed under argon for 24 h. The resulting mixture was poured into water and extracted with chloroform. The combined organic layer was washed with water, and dried over Na_2_SO_4_. After removed the solvent under reduced pressure, the residue was purified with a silicone gel column using hexane/chloroform (1:3, v/v) as eluent to obtain the product (yield: 73%).  ^1^H NMR (300 MHz, CDCl_3_, *δ*): 7.73-7.65 (m, 8H), 7.60-7.51 (m, 12H), 7.27 (s, 6H), 3.19 (t, *J* = 6.9 Hz, 8H), 1.98-2.02 (m, 16H), 1.61-1.54 (m, 8H), 1.18-1.02 (m, 44H), 0.80-0.65 (m, 24H). ^13^C NMR (75 MHz, CDCl_3_, *δ*): 152.08, 151.34, 150.80, 141.23, 141.01, 140.66, 139.93, 130.92, 127.38, 127.21, 126.49, 126.25, 123.06, 121.86, 121.50, 120.53, 120.28, 120.06, 91.11, 55.54, 55.29, 40.70, 40.47, 34.12, 32.85, 31.78, 29.97, 29.28, 27.98, 24.05, 23.83, 22.87, 14.35. MS (MALDI-TOF, m/z): [M]^+^ Calcd for C_102_H_126_Br_4_: 1670.7; Found: 1671.5; Anal. Calcd for C_102_H_126_Br_4_: C 73.28, H 7.60; Found: C 73.33, H 7.26.

***1,2,3,4,5,6-hexakis(9',9'-bis(6-bromohexyl)-9,9-dihexyl-9H,9'H-[2,2'-bifluoren]-7-yl)benzene (5).*** To a mixture of 4 (1.04 g, 0.6 mmol) and dicobaltoctacarbonyl (10 mg, 0.03 mmol) was added degassed 1,4-dioxane (12 mL). The solution was refluxed under argon for 24 h. The resulting mixture was poured into water, and the solid was collected by filtration. Then the crude product was purified by column chromatography using hexane/chloroform (3:1, v/v) as eluent to obtain the product as white powder (yield: 62%).  ^1^H NMR (300 MHz, CDCl_3_, *δ*): 7.71-7.69 (m, 12H), 7.58-7.54 (m, 12H), 7.36-7.34 (m, 18H), 3.29 (t, *J* = 6.9 Hz, 24H), 2.00 (t, *J* = 8.4 Hz, 24H), 1.71-1.61 (m, 24H), 1.24-1.13 (m, 24H), 1.10-1.03 (m, 24H), 0.64-0.58 (m, 24H). ^13^C NMR (75 MHz, CDCl_3_, *δ*): 151.54, 151.15, 150.76, 149.81, 141.33, 141.03, 140.48, 140.27, 140.01, 138.15, 129.64, 127.21, 127.13, 126.66, 126.28, 125.96, 123.00, 121.45, 121.25, 120.07, 119.96, 118.16, 55.19, 54.98, 40.77, 40.40, 34.11, 32.82, 31.79, 30.15, 29.24, 27.93, 23.76, 23.07, 22.93, 14.42. MS (MALDI-TOF, m/z): [M]^+^ Calcd for C_306_H_378_Br_12_: 5015.0; Found: 5016.0; Anal. Calcd for C_306_H_378_Br_12_: C 73.28, H 7.60; Found: C 73.62, H 7.83.

***1,2,3,4,5,6-hexakis[9,9-bis(6-(diethanolamino)hexyl)-9H-fluoren-2-yl]benzene (T0-OH).*** Diethanolamine (0.5 g) was added to a solution of 3 (100 mg) in a mixture of tetrahydrofuran (10 mL) and DMF (10 mL). The mixture was stirred vigorously for 5 days at 60℃. The reaction mixture was dialyzed in deionized water for 3 days. Solvent was removed and the obtained solid was dried under reduced pressure overnight to obtain the final product as colorless solid (yield 82%). ^1^H NMR (300 MHz, CD_3_OD, *δ*): 7.30-6.94 (m, 42H), 4.88 (s, 24H), 3.56-3.54 (48H), 2.57-2.55 (m, 48H), 2.30-2.28 (m, 24H), 1.84 (br, 24H), 1.19 (br, 24H), 0.87 (br, 48H), 0.14 (br, 24H). ^13^C NMR (75 MHz, CD_3_OD, *δ*): 152.01, 150.44, 142.46, 141.62, 139.89, 130.98, 128.07, 127.52, 123.81, 120.77, 118.89, 60.80, 57.72, 56.45, 56.06, 41.81, 31.36, 28.44, 27.88, 24.76; MS (MALDI-TOF, m/z): [M]^+^ Calcd for C_204_H_306_N_12_O_24_: 3310.3; Found: 3144.0. Anal. Calcd for C_204_H_306_N_12_O_24_: C 74.01, H 9.32, N 5.08. Found: C 74.37, H 9.35, N 4.87.

***1,2,3,4,5,6-hexakis(9',9'-(6-(diethanolamino)hexyl)-9,9-dihexyl-9H,9'H-[2,2'-bifluoren]-7-yl)benzene (T1-OH)***. Prepared as a white solid according to a similar procedure to T0-OH, from 5 and diethanolamine. ^1^H NMR (300 MHz, CD_3_OD, *δ*): 7.91-7.79 (m, 12H), 7.68-7.58 (m, 12H), 7.39-7.34 (m, 18H), 3.25 (t, *J* = 6.9 Hz, 24H), 1.98 (t, *J* = 8.4 Hz, 24H), 1.75-1.66 (m, 24H), 1.29-1.17 (m, 24H), 1.14-1.03 (m, 24H), 0.68-0.59 (m, 24H). ^13^C NMR (75 MHz, CD_3_OD, *δ*): 151.65, 151.43, 149.97, 144.41, 142.07, 141.45, 141.28, 140.23, 139.25, 128.70, 127.10, 126.87, 126.65, 126.23, 125.96, 123.32, 121.32, 121.25, 119.96, 119.78, 118.36, 57.31, 54.00, 41.79, 40.52, 31.80, 31.95, 30.57, 28.86, 23.69, 23.33, 23.07, 22.65, 14.36, 14.20. Yield: 86%. MS (MALDI-TOF, m/z): [M]^+^ Calcd for C_354_H_498_N_12_O_24_: 5304.8. Found: 5306.0; Anal. Calcd for C_354_H_498_N_12_O_24_: C 80.13, H 9.46, N 3.17; Found: C 79.89, H 9.60, N 2.97.

**Figure S2.** a) Normalized UV-vis absorption spectra of T0-OH and T1-OH in methanol solution and in solid-state films and b) cyclic voltammograms of T0-OH and T1-OH in THF solution for oxidation.

**Table S1.** The absorption data and HOMO/LUMO data for T0-OH and T1-OH

| Molecule | λ_abs_^a^ [nm] | λ_abs_, _film_ [nm] | *E*_g_^b^  [eV] | *E*_ox_^c^  [V] | HOMO^d^  [eV] | LUMO^e^  [eV] |
| --- | --- | --- | --- | --- | --- | --- |
| T0-OH | 315 | 315 | 3.65 | 0.92, 1.12, 1.30 | -5.72 | -2.07 |
| T1-OH | 342 | 342 | 3.30 | 0.86, 1.23 | -5.65 | -2.35 |

*^a^* Measured for 1 x 10^-6^ M methanol solutions. *^b^* Caculated from the edge of low-energy absorption edge. *^c^* Recorded at scan rate = 100 mV s^-1^; *E* (ferrocene) = 0.53 V versus Ag/AgCl reference electrode. *^d^* Determined from the onset of oxidation potentials. *^e^* Deduced from HOMO and *E*_g_ estimated from the red edge of the longest absorption wavelength for the solid-film sample.

1 Lee, S. H., Nakamura, T. & Tsutsui, T. Synthesis and Characterization of Oligo(9,9-dihexyl-2,7-fluorene ethynylene)s:  For Application as Blue Light-Emitting Diode. *Org. Lett.* **3**, 2005-2007, (2001).

2 Zou, Y., Ye, T., Ma, D., Qin, J. & Yang, C. Star-shaped hexakis(9,9-dihexyl-9H-fluoren-2-yl)benzene end-capped with carbazole and diphenylamine units: solution-processable, high Tg hole-transporting materials for organic light-emitting devices. *J. Mater. Chem.* **22**, 23485-23491, (2012).

3 Liu, B., Dan, T. T. T. & Bazan, G. C. Collective Response from a Cationic Tetrahedral Fluorene for Label-Free DNA Detection. *Adv. Funct. Mater.* **17**, 2432-2438, (2007).
